# Supplementary material for: M6A reduction relieves FUS-associated ALS granules
Source: Nat Commun. 2024 Jun 12;15:5033. doi: 10.1038/s41467-024-49416-5 (PMC11169559; doi:10.1038/s41467-024-49416-5)
Supplement: Supplementary file 1 — Supplementary Information [file 41467_2024_49416_MOESM1_ESM.pdf]

**Supplementary information**

## M$^6$A reduction relieves FUS-associated ALS granules

Gaia Di Timoteo[1, #], Andrea Giuliani[1, #], Adriano Setti[1], Martina C. Biagi[2], Michela Lisi[1], Tiziana Santini[1], Alessia Grandioso[1], Davide Mariani[3], Francesco Castagnetti[3], Eleonora Perego[3], Sabrina Zappone[3], Serena Lattante[4], Mario Sabatelli[5-6], Dante Rotili[7], Giuseppe Vicidomini[3] and Irene Bozzoni[*1,2,3]

[1] Department of Biology and Biotechnology Charles Darwin, Sapienza University of Rome, Rome 00185, Italy.

[2] Center for Life Nano- & Neuro-Science@Sapienza, Fondazione Istituto Italiano di Tecnologia (IIT), Rome 00161, Italy.

[3] Center for Human Technologies@Istituto Italiano di Tecnologia (IIT), Genoa 16152, Italy.

[4] Section of Genomic Medicine, Department of Life Sciences and Public Health, Università Cattolica del Sacro Cuore, 00168 Rome, Italy

[5] Section of Neurology, Department of Neuroscience, Faculty of Medicine and Surgery, Università Cattolica del Sacro Cuore, 00168 Rome, Italy

[6] Adult NEMO Clinical Center, Unit of Neurology, Department of Aging, Neurological, Orthopedic and Head-Neck Sciences, Fondazione Policlinico Universitario A. Gemelli IRCCS, 00168 Rome, Italy

[7] Department of Drug Chemistry and Technologies, Sapienza University of Rome, 00185 Rome, Italy

[#] These authors contributed equally: Gaia Di Timoteo, Andrea Giuliani

* Corresponding author. Email: irene.bozzoni@uniroma1.it

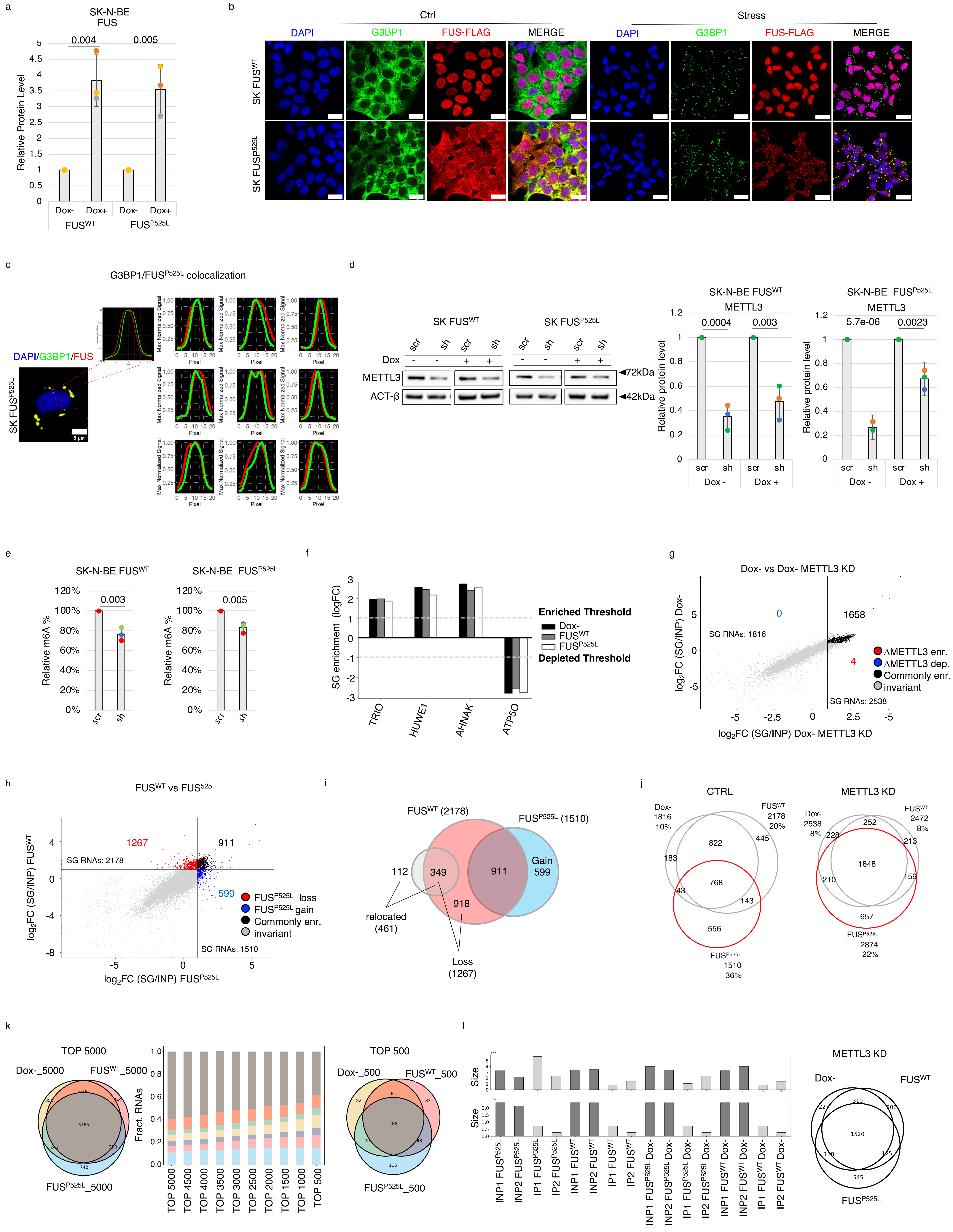
a
SK-N-BE FUS
Relative Protein Level
0.004
0.005
Dox- Dox+ Dox- Dox+
FUSWT FUSP525L
b
Ctrl
Stress
DAPI G3BP1 FUS-FLAG MERGE
SK FUSWT
SK FUSP525L
c
G3BP1/FUSP525L colocalization
DAPI/G3BP1/FUS
SK FUSP525L
5 µm
Max Normalized Signal
Pixel
d
SK FUSWT
SK FUSP525L
scr sh scr sh
Dox - - + +
METTL3
ACT-β
◂72kDa
◂42kDa
SK-N-BE FUSWT
METTL3
0.0004
0.003
SK-N-BE FUSP525L
METTL3
5.7e-06
0.0023
Relative protein level
scr sh scr sh
Dox - Dox +
e
SK-N-BE FUSWT
SK-N-BE FUSP525L
Relative m6A %
0.003
0.005
scr sh
f
SG enrichment (logFC)
Enriched Threshold
Dox-
FUSWT
FUSP525L
Depleted Threshold
TRIO
HUWE1
AHNAK
ATP5O
g
Dox- vs Dox- METTL3 KD
0
1658
SG RNAs: 1816
4
SG RNAs: 2538
ΔMETTL3 enr.
ΔMETTL3 dep.
Commonly enr.
invariant
log2FC (SG/INP) Dox-
log2FC (SG/INP) Dox- METTL3 KD
h
FUSWT vs FUS525
1267
911
SG RNAs: 2178
599
SG RNAs: 1510
FUSP525L loss
FUSP525L gain
Commonly enr.
invariant
log2FC (SG/INP) FUSWT
log2FC (SG/INP) FUSP525L
i
FUSWT (2178)
FUSP525L (1510)
112
349
911
Gain 599
918
relocated (461)
Loss (1267)
j
CTRL
Dox- 1816 10%
FUSWT 2178 20%
822
445
183
768
43
143
556
FUSP525L 1510 36%
METTL3 KD
Dox- 2538 8%
FUSWT 2472 8%
252
228
213
1848
210
159
657
FUSP525L 2874 22%
k
TOP 5000
Dox-_5000
FUSWT_5000
FUSP525L_5000
Fract. RNAs
TOP 5000 TOP 4500 TOP 4000 TOP 3500 TOP 3000 TOP 2500 TOP 2000 TOP 1500 TOP 1000 TOP 500
TOP 500
Dox-_500
FUSWT_500
FUSP525L_500
l
Size
INP1 FUSP525L INP2 FUSP525L IP1 FUSP525L IP2 FUSP525L INP1 FUSWT INP2 FUSWT IP1 FUSWT IP2 FUSWT INP1 FUSP525L Dox- INP2 FUSP525L Dox- IP1 FUSP525L Dox- IP2 FUSP525L Dox- INP1 FUSWT Dox- INP2 FUSWT Dox- IP1 FUSWT Dox- IP2 FUSWT Dox-
METTL3 KD
Dox-
FUSWT
310
227
206
1520
138
125
545
FUSP525L

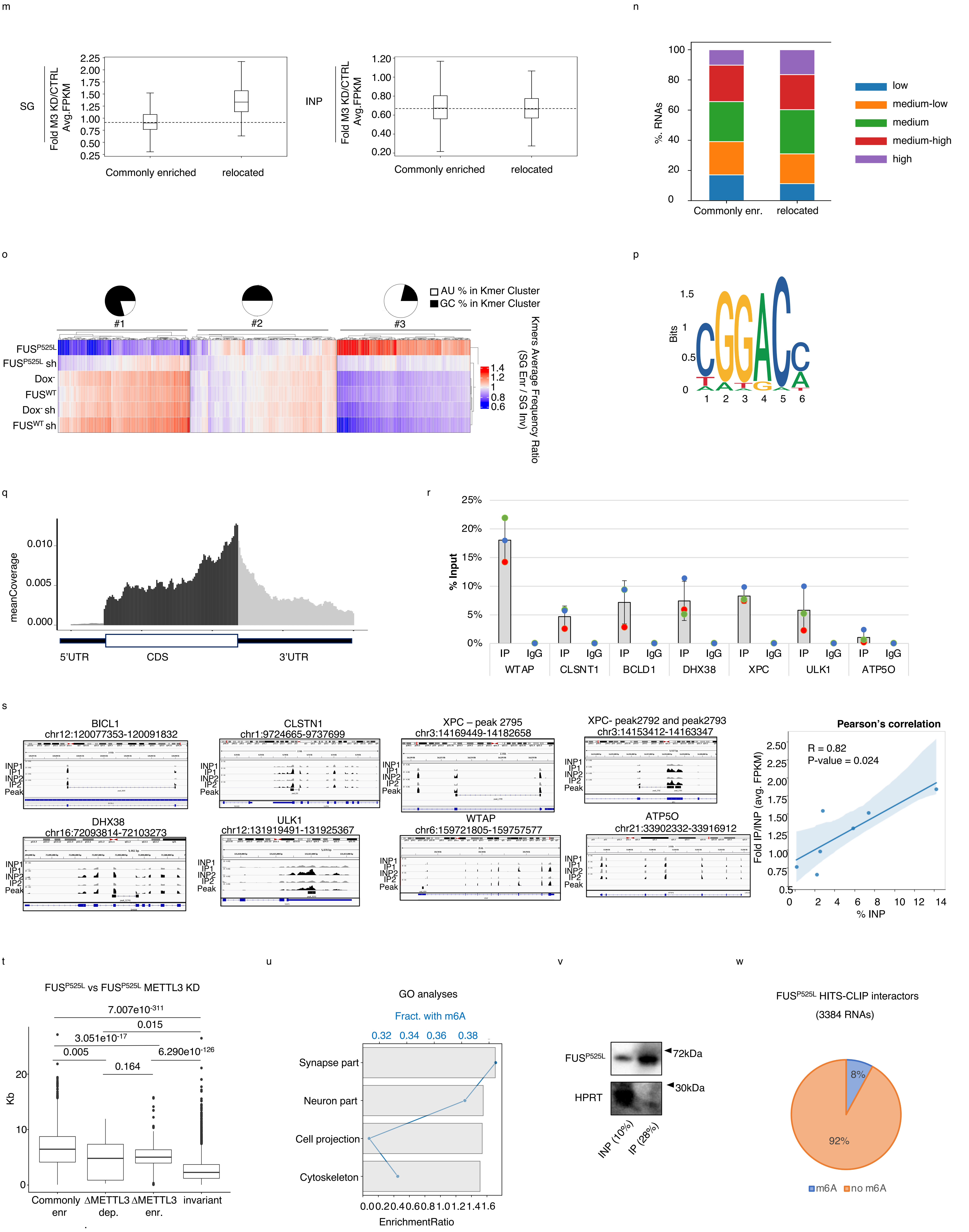
m
SG
Fold M3 KD/CTRL Avg.FPKM
2.25
2.00
1.75
1.50
1.25
1.00
0.75
0.50
0.25
Commonly enriched
relocated
INP
Fold M3 KD/CTRL Avg.FPKM
1.20
1.00
0.80
0.60
0.40
0.20
Commonly enriched
relocated
n
100
80
60
40
20
0
%. RNAs
Commonly enr.
relocated
low
medium-low
medium
medium-high
high
o
AU % in Kmer Cluster
GC % in Kmer Cluster
#1
#2
#3
FUS^P525L
FUS^P525L sh
Dox^-
FUS^WT
Dox^- sh
FUS^WT sh
1.4
1.2
1
0.8
0.6
Kmers Average Frequency Ratio (SG Enr / SG Inv)
p
1.5
1
0.5
0
Bits
1
2
3
4
5
6
q
meanCoverage
0.010
0.005
0.000
5'UTR
CDS
3'UTR
r
25%
20%
15%
10%
5%
0%
% Input
IP
IgG
WTAP
CLSNT1
BCLD1
DHX38
XPC
ULK1
ATP5O
s
BICL1
chr12:120077353-120091832
CLSTN1
chr1:9724665-9737699
XPC – peak 2795
chr3:14169449-14182658
XPC- peak2792 and peak2793
chr3:14153412-14163347
INP1
IP1
INP2
IP2
Peak
DHX38
chr16:72093814-72103273
ULK1
chr12:131919491-131925367
WTAP
chr6:159721805-159757577
ATP5O
chr21:33902332-33916912
Pearson's correlation
R = 0.82
P-value = 0.024
Fold IP/INP (avg. FPKM)
2.50
2.25
2.00
1.75
1.50
1.25
1.00
0.75
0.5
0
2
4
6
8
10
12
14
% INP
t
FUS^P525L vs FUS^P525L METTL3 KD
7.007e10^-311
0.015
3.051e10^-17
0.005
6.290e10^-126
0.164
Kb
20
10
0
Commonly enr
ΔMETTL3 dep.
ΔMETTL3 enr.
invariant
u
GO analyses
Fract. with m6A
0.32
0.34
0.36
0.38
Synapse part
Neuron part
Cell projection
Cytoskeleton
0.00.20.40.60.81.01.21.41.6
EnrichmentRatio
v
FUS^P525L
72kDa
HPRT
30kDa
INP (10%)
IP (28%)
w
FUS^P525L HITS-CLIP interactors
(3384 RNAs)
8%
92%
m6A
no m6A

x

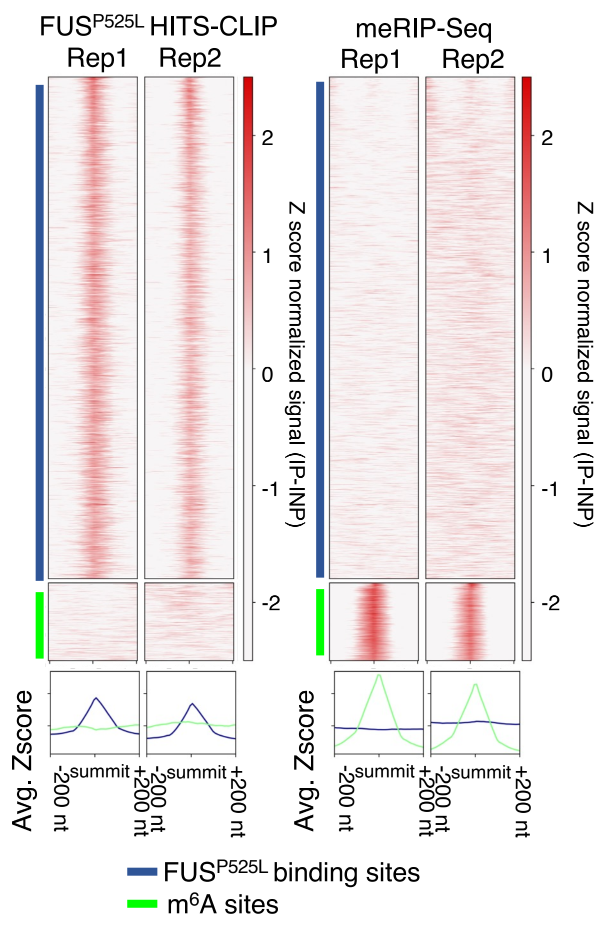


y

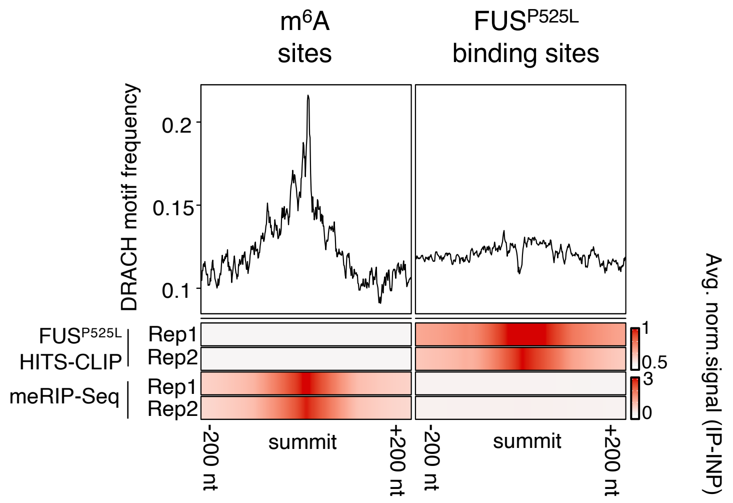


Supplementary Figure 1. **METTL3 downregulation restores the physiological RNA composition of stress granules in ALS cellular models. a** Densitometry analyses showing the relative protein level of FUS in the indicated SK-N-BE cells upon doxycycline induction (Dox+) with respect to the control condition (Dox-). The relative protein quantity in the bars is represented as mean of replicates with standard deviation. Dots represent independent replicates. n=3 biologically independent replicates. The *ratio* of each sample *versus* its experimental control was tested by two-tailed Student's t test. P-values are indicated. **b** Representative images of the indicated SK-N-BE cells either stressed (Stress) or in control (Ctrl) condition. G3BP1 antibody staining is depicted in green, FUS in red. The nuclei are stained with DAPI (blue). The scale bar is 20$\mu$m. **c** Representative image of a SK-N-BE FUS$^{P525L}$ cells in stress condition (left) used for the signal profile analyses of ten granules (right). The nucleus is stained with DAPI (blue). G3BP1 antibody staining, and the corresponding signal is depicted in green, FUS$^{P525L}$ in red. The scale bar is 5$\mu$m. n=10. **d** Representative western blots (left) and the corresponding densitometry analyses (right) evaluating the decrease of METTL3 protein upon shRNAs ("sh") transfection in SK-N-BE FUS$^{WT}$ or SK-N-BE FUS$^{P525L}$ cell lines; ACT-β was used as loading control. Relative protein levels were represented as relative quantities with respect to wild-type cells set to 1. The relative protein quantity in the bars is represented as mean of replicates with standard deviation. Dots represent independent replicates. n=3 biologically independent replicates. The *ratio* of each sample *versus* its experimental control was tested by two-tailed Student's t test. P-values are indicated. **e** Relative quantification of m$^6$A on RNA upon METTL3 knock-down ("sh") with respect to a control condition ("scr", set as 100%) in either FUS$^{WT}$ or FUS$^{P525L}$ SK-N-BE cells. The relative m$^6$A percentage in the bars is represented as mean of replicates with standard deviation. Dots represent independent replicates. n=3 biologically independent replicates. The *ratio* of each sample *versus* its experimental control was tested by two-tailed Student's t test. P-values are indicated. **f** Bar plot depicting SG enrichment ($log_2$FC SG/INP) of selected RNAs to confirm proper SG purification in Dox- (black bars), FUS$^{WT}$ (grey bars) and FUS$^{P525L}$ (white bars) conditions: HUWE1, TRIO and AHNAK were used as positive controls while ATP5O as negative one. n=2 biologically independent replicates. The $log_2$FC thresholds to define SG enriched RNAs ($log_2$FC>1) and depleted ones ($log_2$FC<-1) are indicated as dotted grey lines. **g** Scatter plot depicting RNA differential enrichment in SG in FUS$^{P525L}$ *vs* FUS$^{P525L}$ METTL3-KD condition. Axes describe $log_2$FC of SG RNA enrichment in the indicated conditions. Red dots indicate *ΔMETTL3-enriched* RNAs. Blue dots indicate *ΔMETTL3-depleted* RNAs. Black dots indicate *commonly enriched* RNAs that are enriched in SG in both conditions. Grey dots indicate *invariant* RNAs. n=2 biologically independent replicates. **h** Scatter plot depicting RNA differential enrichment in FUS$^{WT}$ vs FUS$^{P525L}$. Axes describe $log_2$FC of SG RNA enrichment in the indicated conditions. Red dots indicate FUS$^{P525}$ *loss* RNAs. Blue dots indicate FUS$^{P525}$ *gain* RNAs. Black dots indicate *commonly enriched* RNAs that are enriched in SG in both conditions. Grey dots indicate *invariant* RNAs. n=2 biologically independent replicates. **i** Venn diagram depicting the overlap between *ΔMETTL3-relocated* RNAs and SG enriched RNAs in FUS$^{WT}$ and FUS$^{P525L}$ conditions. **j** Venn diagrams depicting the overlap among the SG enriched RNAs in the three analyzed conditions (Dox-, FUS$^{WT}$ and FUS$^{P525L}$) in control cells (left panel) and upon METTL3 downregulation (METTL3 KD, right panel). **k** Venn diagrams depicting the overlap among the top 5000 (left) and top 500 RNAs (right) ranked by SG enrichment in Dox-, FUS$^{WT}$ and FUS$^{P525L}$ condition upon METTL3 depletion. Bar plot (middle panel) displaying the fraction of RNAs commonly or specifically enriched in SG in each experimental condition. Different numbers of top ranked RNAs were considered. The different fractions are reported using colors coherent with adjacent Venn diagrams. **l** Bar plot depicting the library sizes of RNA-Seq samples before (top) and after the resampling procedure (bottom). Library sizes of FUS$^{P525L}$ samples were used as reference for resampling (left). Venn diagram (right) displaying the overlap between SG enriched RNAs in the three analyzed conditions (Dox-, FUS$^{WT}$ and FUS $^{P525L}$ upon METTL3 depletion) after the resampling procedure. **m** Box plots displaying the fold of METTL3 knockdown ("M3 KD") over control condition ("CTRL") average abundance (FPKM) for the *commonly enriched* and r*elocated* group either in SG fraction (left panel) or INP (right panel) samples. Dotted line represents the median fold distribution of the *commonly enriched* group, used as control. n=2 biologically independent replicates. **n** Stacked bar plot showing the percentage of *commonly enriched*, *ΔMETTL3-enriched*, *invariant* transcripts in each expression-based transcriptome *stratum*. Each *stratum* is depicted with a s specific color. n=2 biologically independent replicates. **o** Heatmap (bottom) showing k-mers (4-mers) enrichment in SG among different condition. Rows represent each possible k-mer with k=4, while columns represent conditions. K-mers enrichment is described as *ratio* between mean k-mer percentage in SG enriched RNAs *vs* invariant RNAs. The three clusters identified using K-means clustering (n clusters=3) indicate k-mers depleted (#1), invariant (#2) or enriched (#3) in FUS$^{P525L}$ condition. Color scale describes the magnitude of k-mer enrichment, enrichment values > 1 are represented in red while enrichment values < 1 are represented in blue. Pie charts (top) represents the fraction of GC and AU nucleotides in k-mers clusters. n=2 biologically independent replicates. **p** Sequence logo representing the consensus DRACH motif significantly over-represented in the MeRIP-seq peak regions. **q** Metagene plot resulted from MeRIP-seq data displaying m$^6$A increased signal intensity near the STOP codon. **r** Bar blot representing the enrichment of selected RNAs resulted methylated according to MeRIP-seq data. WTAP and ATP5O were used as positive and negative controls, respectively; immunoprecipitation with IgG was used as control. n=3 biologically independent replicates. **s** IGV screens (left) describing the normalized read coverage of meRIP-Seq INP and IP samples in correspondence of peak regions of the indicated transcripts. Scatterplot (right) depicting the correlation between meRIP-Seq signal and RNA enrichment expressed as percentage of input resulted from qRT-PCR analysis for each transcript. Pearson's correlation coefficient (R=0.82) and significance (p-value=0.024) are reported in the plot. Y-axis reports the ratio of IP over the INP average signal (FPKM) while x-axis reports the % of Input related to qRT-PCR analysis. Fitted regression model is reported as a blue line. Confidence intervals are depicted as blue bands. **t** Box plots displaying RNA length (Kb) of the indicated RNA groups identified by SG differential enrichment analysis among FUS$^{P525L}$ *vs* FUS$^{P525L}$ METTL3 KD. Statistical significance was evaluated with Mann-Whitney U test. P-values are indicated in the figure. **u** Bar plot depicting the indicated GO over-represented categories in the *relocated* group. Only significant categories (FDR < 0.05) of Cellular Component database were depicted. Blue line describes the fraction of m$^6$A -containing RNAs for each category. Category enrichment score and fraction of methylated RNAs are indicated either in the bottom or top X-axes, respectively. Absolute number of genes for each category is indicated. **v** Representative western blot showing FUS$^{P525L}$ IP efficiency. The percentage of input is indicated. n=2 biologically independent replicates. **w** Pie chart showing the percentage of m$^6$A-containing RNAs among FUS direct interactors. **x** Heatmaps depicting the signal (Z score calculated form the normalized signal (IP-INP) of the replicates) of either FUS$^{P525L}$ HITS-CLIP (left) or meRIP-seq (right) in correspondence of both FUS$^{P525L}$ binding sites (blue) and m$^6$A sites (green). For each experiment we considered a window of ±200nt around the peak summit. The average of the Z scores calculated is summarized in the line plots (bottom). n=2 biologically independent replicates. **y** Line plots depicting DRACH motif frequency on either m6A sites (left) or FUS$^{P525L}$ binding sites (right). The heatmaps depict the average normalized signal of either FUS$^{P525L}$ HITS-CLIP or meRIP-seq in a window of ±200nt around the peak summit. n=2 biologically independent replicates.

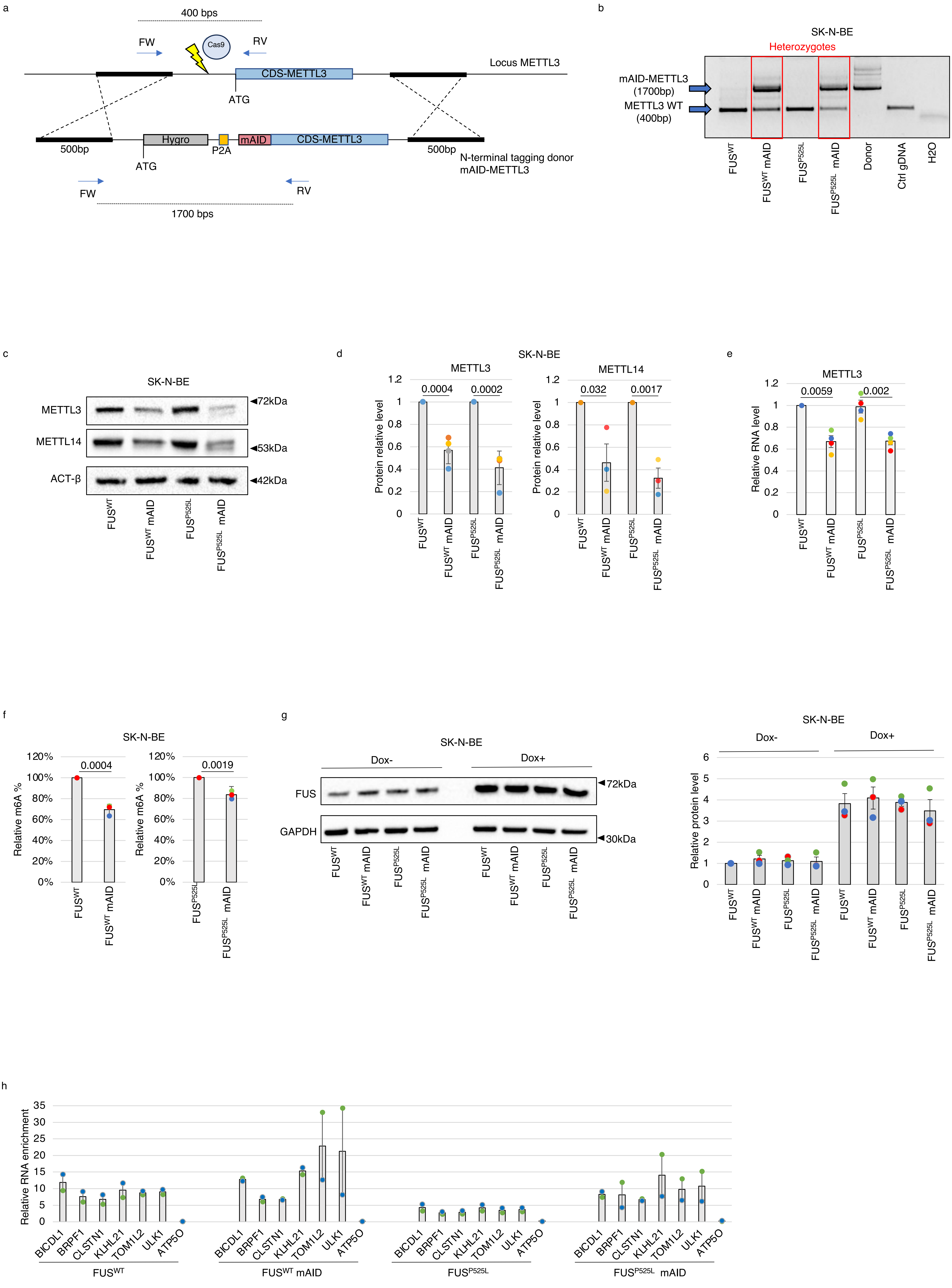

a
400 bps
FW
Cas9
RV
CDS-METTL3
Locus METTL3
ATG
Hygro
mAID
CDS-METTL3
500bp
P2A
500bp
N-terminal tagging donor
mAID-METTL3
ATG
FW
RV
1700 bps
b
SK-N-BE
Heterozygotes
mAID-METTL3 (1700bp)
METTL3 WT (400bp)
FUS$^{WT}$
FUS$^{WT}$ mAID
FUS$^{P525L}$
FUS$^{P525L}$ mAID
Donor
Ctrl gDNA
H2O
c
SK-N-BE
METTL3
METTL14
ACT-β
◀72kDa
◀53kDa
◀42kDa
d
SK-N-BE
METTL3
METTL14
Protein relative level
0.0004
0.0002
0.032
0.0017
e
METTL3
Relative RNA level
0.0059
0.002
f
SK-N-BE
Relative m6A %
0.0004
0.0019
g
SK-N-BE
Dox-
Dox+
FUS
GAPDH
◀72kDa
◀30kDa
Relative protein level
h
Relative RNA enrichment
BICDL1
BRPF1
CLSTN1
KLHL21
TOM1L2
ULK1
ATP5O
FUS$^{WT}$
FUS$^{WT}$ mAID
FUS$^{P525L}$
FUS$^{P525L}$ mAID

i

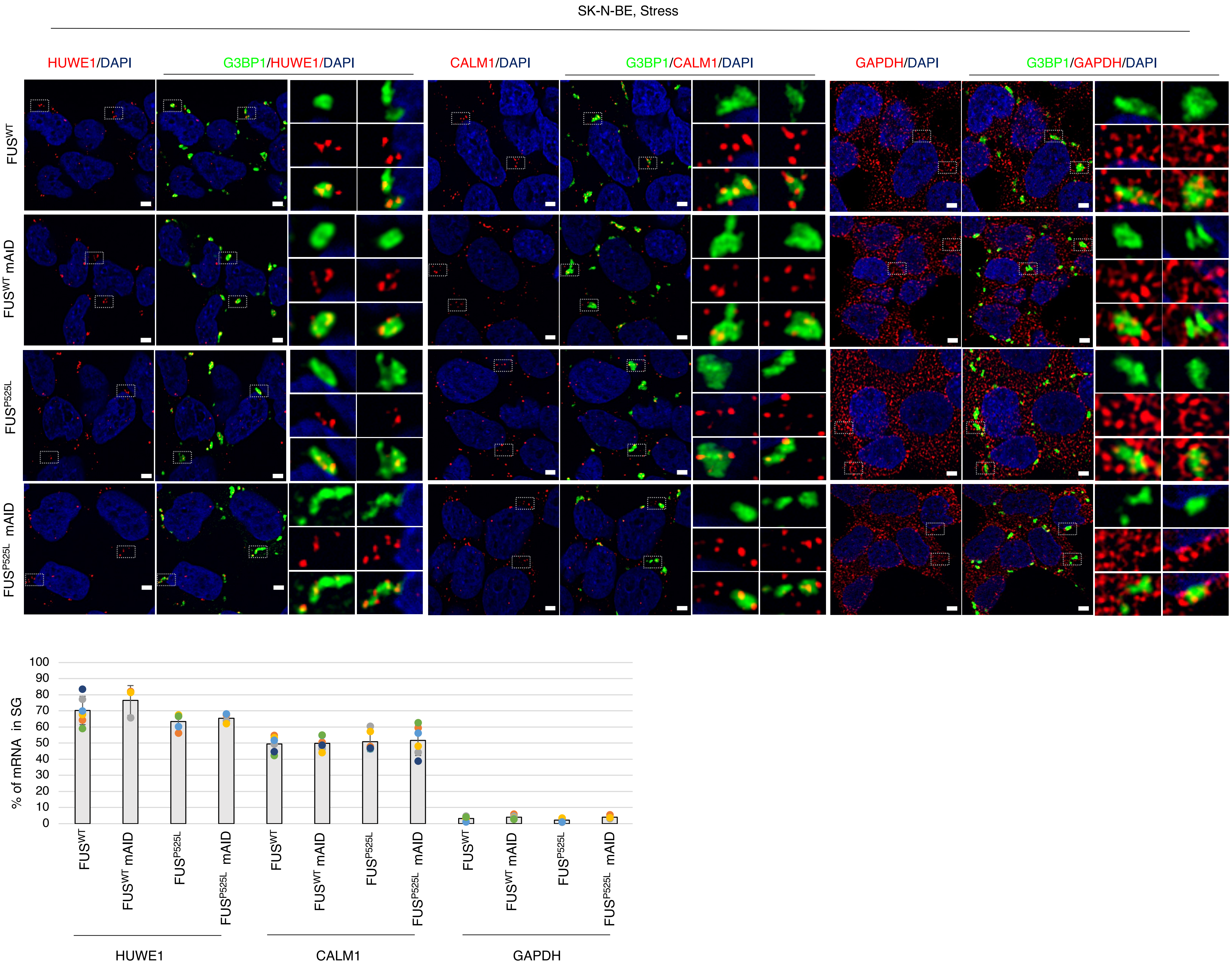
SK-N-BE, Stress
HUWE1/DAPI
G3BP1/HUWE1/DAPI
CALM1/DAPI
G3BP1/CALM1/DAPI
GAPDH/DAPI
G3BP1/GAPDH/DAPI
FUS^WT
FUS^WT mAID
FUS^P525L
FUS^P525L mAID
% of mRNA in SG
100
90
80
70
60
50
40
30
20
10
0
HUWE1
CALM1
GAPDH

j

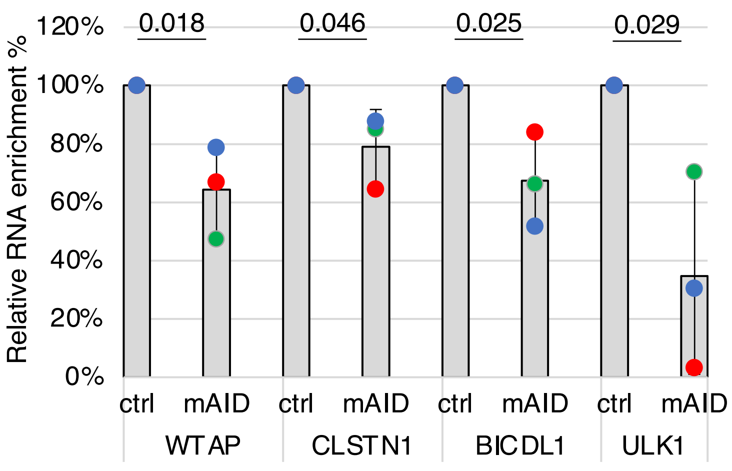
Relative RNA enrichment %
120%
100%
80%
60%
40%
20%
0%
0.018
0.046
0.025
0.029
ctrl
mAID
WTAP
CLSTN1
BICDL1
ULK1

k

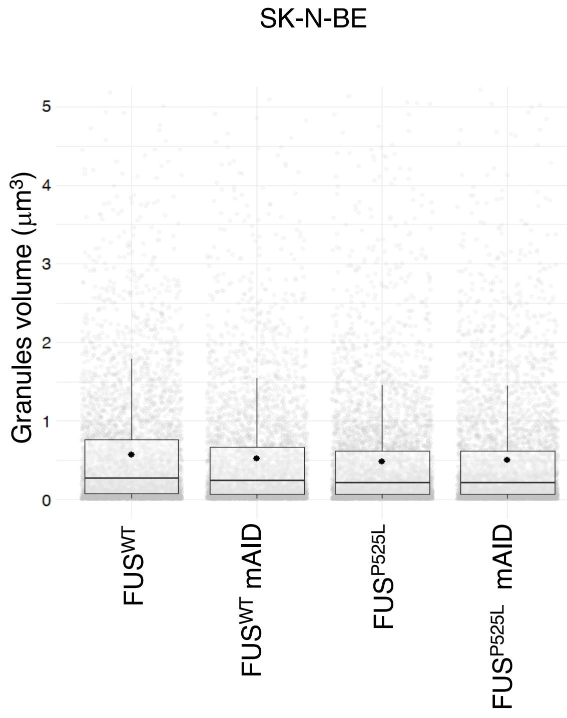
SK-N-BE
Granules volume (µm³)
5
4
3
2
1
0
FUS^WT
FUS^WT mAID
FUS^P525L
FUS^P525L mAID

l

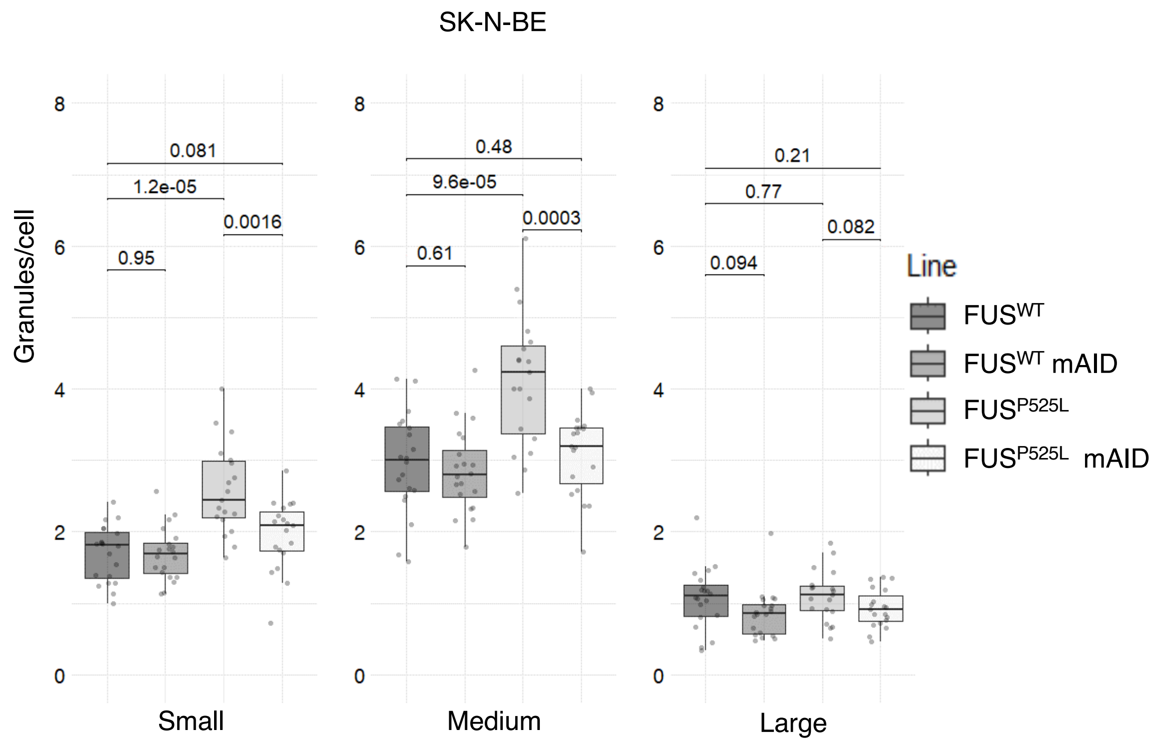
SK-N-BE
Granules/cell
8
6
4
2
0
0.081
1.2e-05
0.95
0.0016
0.48
9.6e-05
0.61
0.0003
0.21
0.77
0.094
0.082
Small
Medium
Large
Line
FUS^WT
FUS^WT mAID
FUS^P525L
FUS^P525L mAID

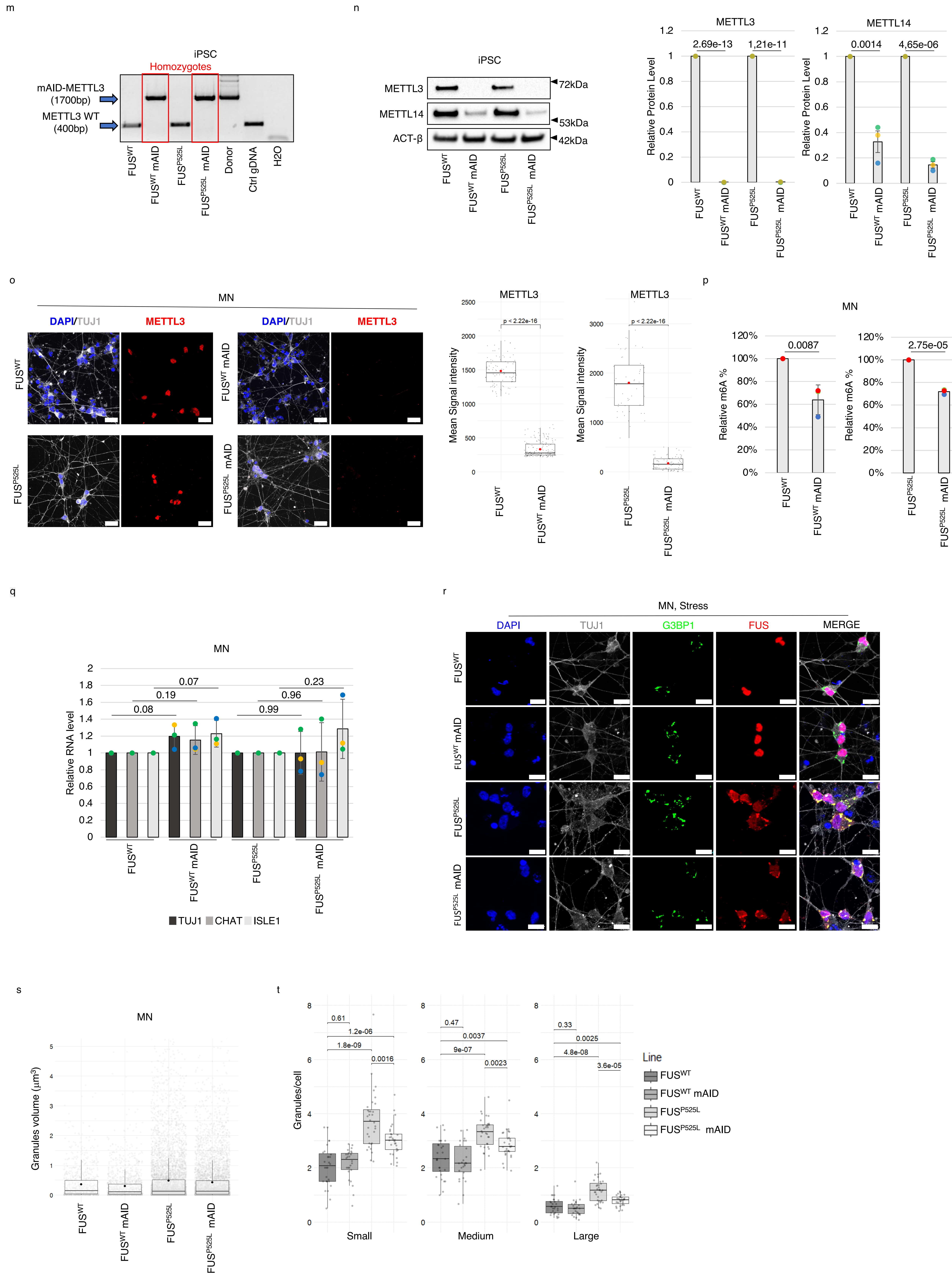

m
iPSC
Homozygotes
mAID-METTL3 (1700bp)
METTL3 WT (400bp)
FUS^WT
FUS^WT mAID
FUS^P525L
FUS^P525L mAID
Donor
Ctrl gDNA
H2O
n
iPSC
METTL3
METTL14
ACT-β
72kDa
53kDa
42kDa
METTL3
METTL14
2.69e-13
1,21e-11
0.0014
4,65e-06
Relative Protein Level
o
MN
DAPI/TUJ1
METTL3
Mean Signal intensity
p < 2.22e-16
p
MN
Relative m6A %
0.0087
2.75e-05
q
MN
Relative RNA level
0.07
0.19
0.08
0.23
0.96
0.99
TUJ1
CHAT
ISLE1
r
MN, Stress
DAPI
TUJ1
G3BP1
FUS
MERGE
s
MN
Granules volume (µm³)
t
Granules/cell
0.61
1.2e-06
1.8e-09
0.0016
0.47
0.0037
9e-07
0.0023
0.33
0.0025
4.8e-08
3.6e-05
Small
Medium
Large
Line

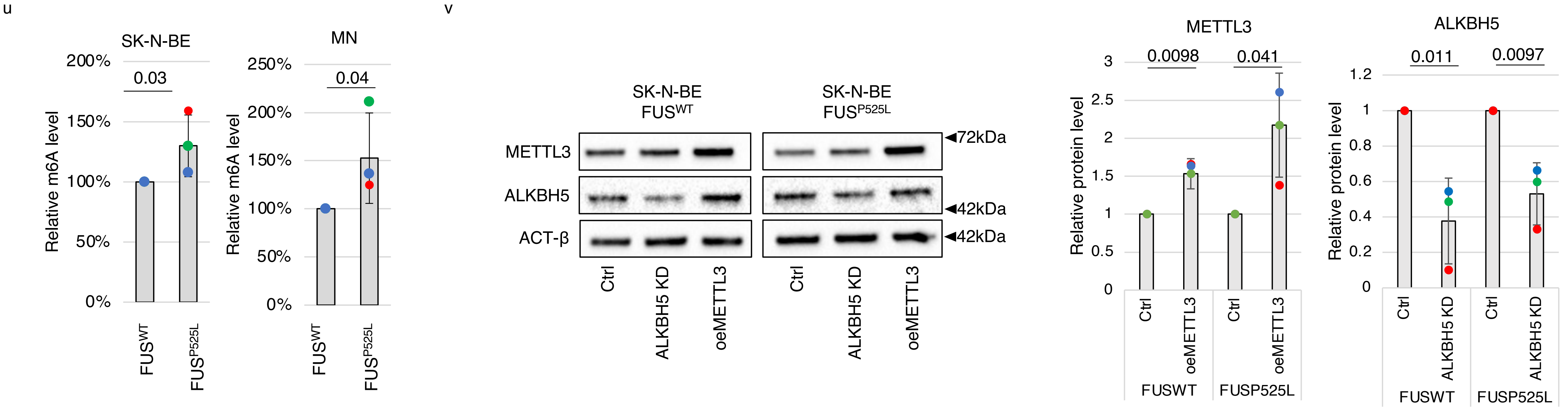


Supplementary Figure 2. **METTL3 downregulation reduces the number of SG in ALS cellular models.** **a** Schematic representation of the METTL3 *locus* and the plasmid donor used for CRISPR/Cas-9 mediated genome editing to insert a degron tag (mAID) at the N-terminus of METTL3. Primers for genotyping are indicated with arrows. The size of the expected amplicon is specified. **b** Genomic analyses of selected SK-N-BE clones after CRISPR/Cas9 mediated genome editing to insert a degron tag (mAID) at the N-terminus of METTL3. Heterozygotes are red squared. Blue arrows indicate the size of the amplicons deriving from either wild type (METTL3 WT) or edited genome (mAID METTL3). The donor DNA and gDNA from unedited cells were used as positive or negative controls, respectively. **c** Representative western blot analysis showing METTL3 and METTL14 decrease in the indicated SK-N-BE cells after degron tag insertion (mAID). ACT-β was used as loading control. **d** Relative protein levels of METTL3 (n=4 biologically independent replicates) and METTL14 (n=3 biologically independent replicates) in the indicated SK-N-BE upon degron tag (mAID) insertion. Levels were normalized over ACT-β protein level and expressed as relative quantities with respect to wild-type cells set to 1. The relative protein quantity in the bars is represented as mean of replicates with standard deviation. Dots represent biologically independent replicates. The *ratio* of each sample *versus* its experimental control was tested by two-tailed Student's t test. P-values are indicated. **e** Bar plot showing METTL3 relative RNA level decrease in the indicated SK-N-BE cells. Values are normalized against ATP5O transcript and expressed as relative quantity with respect to non-edited cells set to a value of 1. The relative RNA quantity in the bars is represented as mean of the fold change with standard deviation. Dots represent each replicate. n=4 biologically independent replicates. The *ratio* of each sample *versus* its experimental control was tested by two-tailed Student's t test. P-values are indicated. **f** Bar plot showing the m$^6$A level decrease in the indicated SK-N-BE cells. Levels are represented as percentage with respect to control cells set as 100%. Each dot represents a single replicate. n=3 biologically independent replicates. The *ratio* of each sample *versus* its experimental control was tested by two-tailed Student's t test. P-values are indicated. **g** Representative western blot analysis (left) and the corresponding densitometry analyses (right) showing stable FUS expression in the indicated SK-N-BE cells. GAPDH was used as loading control. Relative protein levels were represented as relative quantities with respect to FUS$^{WT}$ Dox- cells set as 1. The relative protein quantity in the bars is represented as mean of replicates with standard deviation. Dots represent independent replicates. n=3 biologically independent replicates. The *ratio* of each sample *versus* its experimental control was tested by two-tailed Student's t test. P-values are indicated. **h** Levels of selected RNAs in a representative stress granules immunoprecipitation in SK-N-BE FUS$^{WT}$ or FUS$^{P525L}$ cell lines, induced for the expression of FUS (Dox+), upon 1hr stress treatment. RNA levels are expressed as percentage of input normalized on an invariant RNA enrichment (CALM1). ATP5O transcript was used as negative control. The relative RNA enrichment in the bars is represented as mean of the replicates with standard deviation. Dots represent each replicate. n=2 biologically independent replicates. The *ratio* of each sample *versus* its experimental control was tested by two-tailed Student's t test. P-values are indicated. **i** Representative single focal planes captions of mFISH for HUWE1, CALM1 and GAPDH transcripts (red), immunofluorescence for stress granule marker G3BP1 (green) and DAPI (blue) in the indicated SK-N-BE cells. All scale bars correspond to 3 $\mu$m (top). In right panels, digital magnification of G3BP1 granules highlighted by dashed squares. Bar plots showing the average percentage of the indicated transcripts signals colocalizing with stress granules as mean of replicates with standard deviation (bottom). Dots represent independent replicates. From a minimum of #283 to a maximum of #629 cells were analyzed in each experimental condition. n=3 biologically independent replicates. The *ratio* of each sample versus its experimental control was tested by two-tailed Student's t test. P-values are indicated. **j** Bar plots showing the individual enrichment reduction of selected relocated candidates in a m$^6$A CLIP experiment in mAID condition with respect to the control condition represented as 100%. WTAP was used as positive control. The relative RNA enrichment in the bars is represented as mean of the replicates with standard deviation. Dots represent each replicate. n=3 biologically independent replicates. The *ratio* of each sample *versus* its experimental control was tested by two-tailed Student's t test. P-values are indicated. **k** Box plots showing granule volumes in the indicated SK-N-BE cells. Black dots represent the average volumes. Gray dots represent the volume of each measured granule. n=3 biologically independent replicates. Seven fields were acquired for each biological replicate. **l** Box plots showing the number of small, medium, or large granules per cell in the indicated SK-N-BE cells. Seven fields were acquired for each biological replicate. n=3 biologically independent replicates. The *ratio* of each sample *versus* its experimental control was tested by two-tailed Student's t test. P-values are indicated. **m** Genomic analyses of selected iPS clones after CRISPR/Cas9 mediated genome editing to insert a degron tag (mAID) at the N-terminus of METTL3. Homozygotes are red squared. Blue arrows indicate the size of the amplicons deriving from either wild type (METTL3 WT) or edited genome (mAID METTL3). The donor DNA and gDNA from unedited cells were used as positive or negative controls, respectively. **n** Representative western blot analysis (left) showing METTL3 and METTL14 decrease in the indicated SK-N-BE cells after degron tag insertion (mAID). ACT-β was used as loading control. Relative protein levels (right) of METTL3 (n=4 biologically independent replicates) and METTL14 (n=3 biologically independent replicates) in the indicated SK-N-BE upon degron tag (mAID) insertion. Levels were normalized over ACT-β protein level and expressed as relative quantities with respect to wild-type cells set to 1. The relative protein level in the bars is represented as mean of the replicates with standard deviation. Dots represent biologically independent replicates. The *ratio* of each sample *versus* its experimental control was tested by two-tailed Student's t test. P-values are indicated. **o** Representative images of METTL3 immunofluorescence on the indicated MNs (left). METTL3 antibody staining is depicted in red, TUJ1 in grey. The nuclei are stained with DAPI (blue). The merge of the signals from TUJ1 and DAPI is shown. The scale bar is 10 $\mu$m. Box plots illustrating METTL3 signal intensity analyses (right). Seven fields were acquired. Red dots represent signal average. Each gray dots represents a single cell signal. The *ratio* of each sample *versus* its experimental control was tested by two-tailed Student's t test. P-values are indicated. The scale bar is 20 $\mu$m. **p** Bar plot showing the m$^6$A level decrease in the indicated iPSC derived MN. Levels are represented as percentage with respect to control cells set as 100%. Each dot represents a single replicate. n=3 biologically independent replicates. The relative m$^6$A percentage in the bars is represented as mean of replicates with standard deviation. The *ratio* of each sample *versus* its experimental control was tested by two-tailed Student's t test. P-values are indicated. **Q,** Bar plot showing the relative RNA levels of selected MN differentiation markers in the indicated MN. Values are normalized against GAPDH transcript and expressed as relative quantity with respect to unedited MN set as 1. The relative RNA quantity in the bars is represented as mean of the fold change with standard deviation. Dots represent each replicate. n=3 biologically independent replicates. The *ratio* of each sample *versus* its experimental control was tested by two-tailed Student's t test. P-values are indicated. **r** Representative images of the indicated stressed MNs. G3BP1 antibody staining is depicted in green, FUS in red, TUJ1 in grey. The nuclei are stained with DAPI (blue). The merge of the signal is shown. The scale bar is 10 $\mu$m. **s** Box plots showing granule volumes in the indicated MN. Black dots represent the average volumes. Gray dots represent the volume of each measured granule. n=4 biologically independent replicates. Seven fields were acquired for each biological replicate. **t** Box plots showing number of small, medium, or large granules per cell in the indicated MN. Seven fields were acquired for each biological replicate. n=4 biologically independent replicates. The *ratio* of each sample *versus* its experimental control was tested by two-tailed Student's t test. P-values are indicated. **u** Bar plot showing the m$^6$A level increase both in SK-N-BE and MNs expressing FUS$^{P525L}$. Levels are represented as percentage with respect to control cells set as 100%. Each dot represents a single replicate. n=3 biologically independent replicates. The relative m$^6$A percentage in the bars is represented as mean of the replicates with standard deviation. The *ratio* of each sample *versus* its experimental control was tested by two-tailed Student's t test. P-values are indicated. **v** Representative western blot analysis (left) and the corresponding densitometry analyses (right) showing the levels of either METTL3 or ALKBH5 in the indicated SK-N-BE cells after METTL3 overexpression ("oeMETTL3") or ALKBH5 downregulation ("ALKBH5 KD"). ACT-β or ACTN1 were used as loading control. Relative protein levels were represented as relative quantities with respect to control (Ctrl) cells set as 1. The relative protein quantity in the bars is represented as mean of replicates with standard deviation. Dots represent independent replicates. n=3 biologically independent replicates. The *ratio* of each sample *versus* its experimental control was tested by two-tailed Student's t test. P-values are indicated.

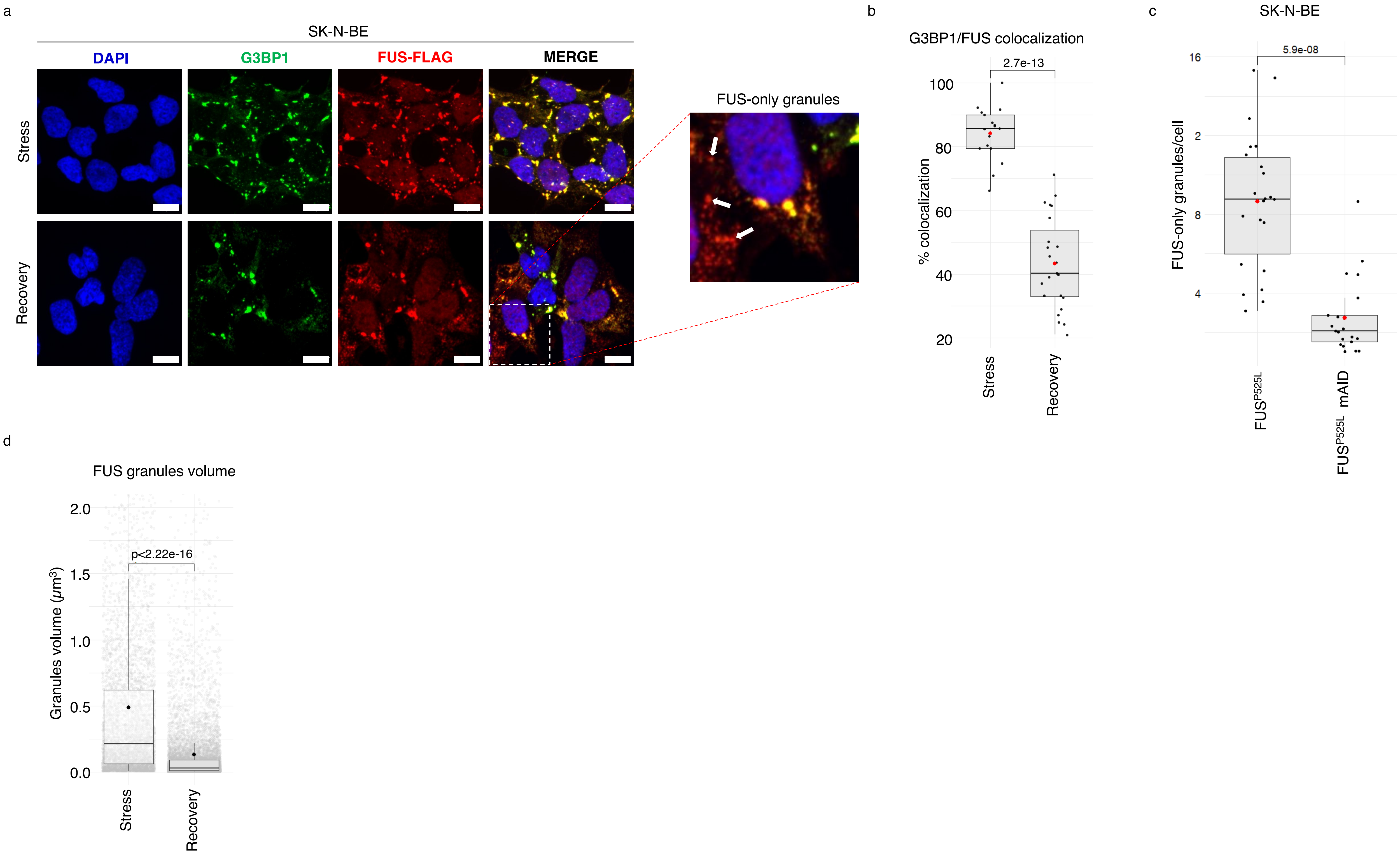


Supplementary Figure 3. **METTL3 decrease restores SG recovery rate in ALS cellular models. a** Representative images of the indicated SK-N-BE cells both in stress and in recovery conditions showing the presence of FUS-only granules indicated by white arrows. G3BP1 antibody staining is depicted in green, FUS in red. The nuclei are stained with DAPI (blue). The merge of the signal is shown. The scale bar is 10 $\mu$m. **b** Box plot showing the colocalization analysis of G3BP1 and FUS signals in SK-N-BE $FUS^{P525L}$ cells both in stress and in recovery conditions. Seven fields were acquired for each biological replicate. n=3 biologically independent replicates. The *ratio* of each sample *versus* its experimental control was tested by two-tailed Student's t test. P-values are indicated. **c** Box plots showing the number of FUS-only granules in the indicated $FUS^{P525L}$ SK-N-BE cells. The red dot indicates the average granules number in each sample. Each dot represents the number of granules in a single field. Seven fields were acquired for each biological replicate. n=4 biologically independent replicates. The *ratio* of each sample *versus* its experimental control was tested by two-tailed Student's t test. P-values are indicated. **d** Box plot showing the comparison of granule volumes in stress or in recovery conditions. Black dots represent the average volumes. Gray dots represent the volume of each measured granule. Seven fields were acquired for each biological replicate. n=3 (stress), n=4 (recovery) biologically independent replicates. The *ratio* of each sample *versus* its experimental control was tested by two-tailed Student's t test. P-values are indicated.

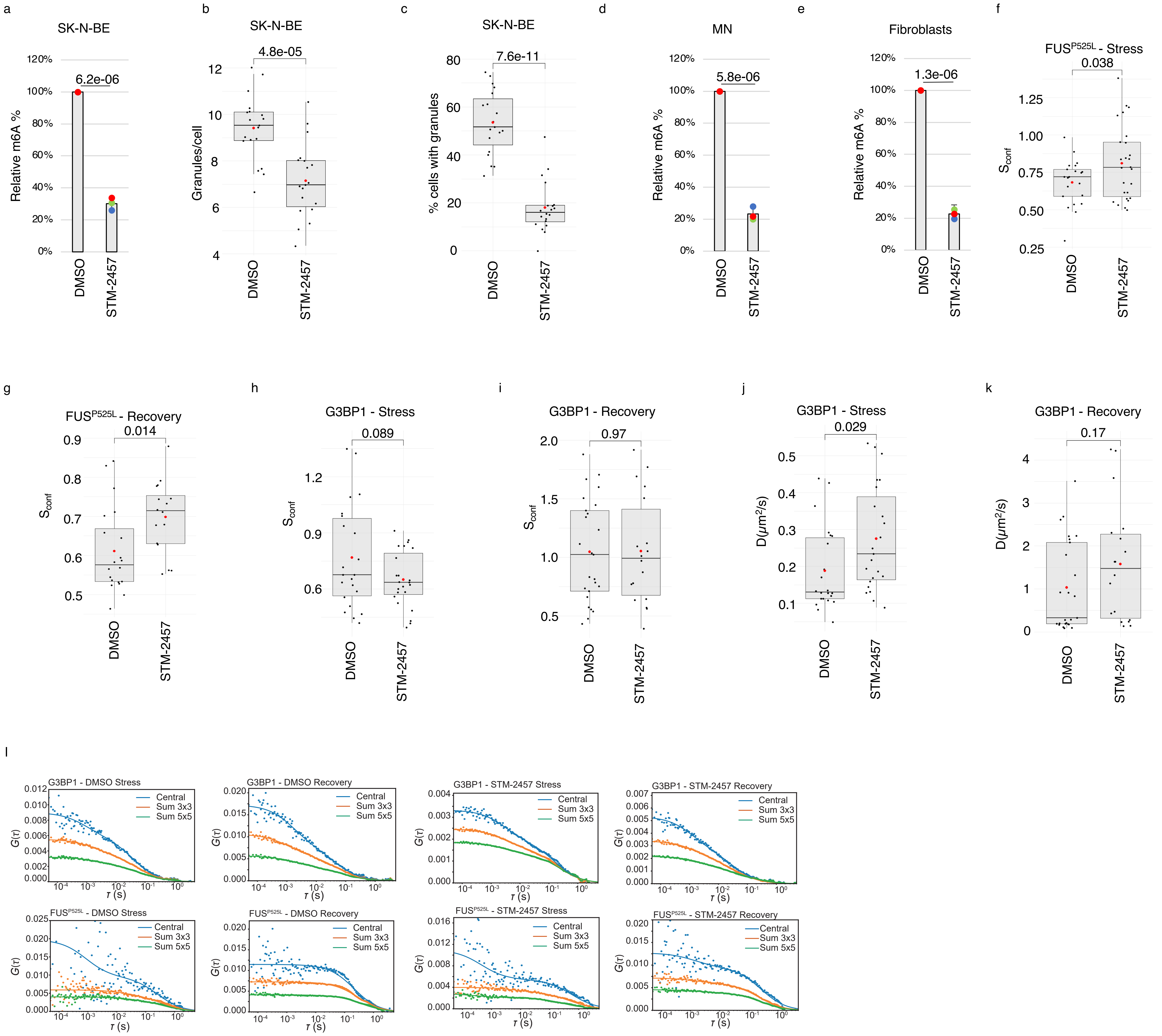


Supplementary Figure 4. **METTL3 chemical inhibition relieves FUS-containing SG. a** Bar plot showing the m$^6$A level decrease of SK-N-BE FUS$^{P525L}$ cells treated either with DMSO or STM-2457. Levels are represented as percentage with respect to control cells set as 100%. Each dot represents a single replicate. n=3 biologically independent replicates. The *ratio* of each sample *versus* its experimental control was tested by two-tailed Student's t test. P-values are indicated. **b** Box plots illustrating the number of stress granules per cell in stressed SK-N-BE FUS$^{P525L}$ treated either with DMSO or STM-2457. The red dot indicates the average number of granules in each sample. Each black dot represents the number of granules in a single field. Seven fields were acquired for each biological replicate. n=3 biologically independent replicates. The *ratio* of each sample *versus* its experimental control was tested by two-tailed Student's t test. P-values are indicated. **c** Box plots showing the percentage of unrecovered SK-N-BE FUS$^{P525L}$ cells treated either with DMSO or STM-2457. The red dot indicates the average percentage in each sample. Each black dot represents the percentage in a single field. Seven fields were acquired for each biological replicate. n=3 biologically independent replicates. The *ratio* of each sample *versus* its experimental control was tested by two-tailed Student's t test. P-values are indicated. **d** Bar plot showing the m$^6$A level decrease of iPSC-derived MN FUS$^{P525L}$ treated either with DMSO or STM-2457. Levels are represented as percentage with respect to control cells set as 100%. Each dot represents a single replicate. n=3 biologically independent replicates. The *ratio* of each sample *versus* its experimental control was tested by two-tailed Student's t test. P-values are indicated. **e** Bar plot showing the m$^6$A level decrease of patient-derived fibroblasts treated either with DMSO or STM-2457. Levels are represented as percentage with respect to control cells set as 100%. Each dot represents a single replicate. n=3 biologically independent replicates. The *ratio* of each sample *versus* its experimental control was tested by two-tailed Student's t test. P-values are indicated. **f, g, h, i, j, k** Box plots illustrating the $S_{conf}$ or D of FUS$^{P525L}$ or G3BP1 in living SK-N-BE cells treated either with DMSO or STM-2457 under stress or recovery condition. The red dot indicates the average value. Each black dot represents a single measurement. n=2 biologically independent replicates. From 16 to 26 measurements were carried out for each experimental condition. The *ratio* of each sample *versus* its experimental control was tested by two-tailed Student's t test. P-values are indicated. **l** Typical spot-variation FCS measurement of G3BP1-GFP (top) or FUS$^{P525L}$ -RFP (bottom) in stress or recovery condition measured in SK-N-BE cells treated either with STM-2457 or DMSO. The three curves correspond to the three volumes considered with the SPAD array detector. The lines correspond to the fit of the autocorrelation curves.
