# Supplementary material for: M6A reduction relieves FUS-associated ALS granules
Source: Nat Commun. 2024 Jun 12;15:5033. doi: 10.1038/s41467-024-49416-5 (PMC11169559; doi:10.1038/s41467-024-49416-5)
Supplement: Supplementary file 3 — Description of Additional Supplementary Files [file 41467_2024_49416_MOESM3_ESM.pdf]

Description of Additional Supplementary Information

File Name: Supplementary_Data _1_RNA-Seq_Summary.xls
Description: Summary of SG enrichment analysis. In the panel "Description" all the information about the other panels is reported. In the panel "Pre-processing", the following columns are reported: reference sample-identifier ("sample_id"); amount of reads in fastq files before pre-processing analysis ("raw_fastq"); amount of reads in fastq files after adapter trimming and reads quality filtering ("trimmed_fastq"); amount of reads after contaminants remotion ("decotaminated_fastq"). In the panel "Alignment_Post-processing", the following columns are reported: reference sample-identifier ("sample_id"); amount of paired-reads used as input for genome (GRCh38) alignment procedure ("Number of input reads"); total amount of fragments mapped to genome ("Total_mapped_reads"); amount of fragments with un-ambiguous mapping position in genome ("Uniquely mapped reads number"); amount of properly-paired reads after deduplication, and mulit-mapping remotion procedures ("Number_of_dedupuplicated_properly-paired_no-multimapped_reads"). In all the other panels the following columns are reported: gene identifier according to Ensembl database (release 99, "gene_id"); average FPKM values after TMM normalization on INPUT samples ("INP"); average FPKM values after TMM normalization on SG-enriched samples ("Sgenr"); log_2fold-change SGenr/INP based on average FPKMs data ("logFC"); average log CPM over all samples of both analyzied conditions ("logCPM"); LR statistics ("LR"); the significance of the differences between conditions ("Pvalue"); multiple-test corrected P-Value according to Benjamini-Hochberg FRD procedure ("FDR"); gene symbol ("external_gene_name"); biotype of gene ("gene_biotype"); label that describe if the RNA analyzed is enriched "enr", invariant "invariant" or depleted ("dep") in SG-enriched samples compared to the matched input ("SG_VS_INP").

File Name: Supplementary_Data _2_m6A-IP.xls
Description: Summary of m6A-IP analysis. In the panel "Description" all the information about the other panels is reported. In the panel "Pre-processing", the following columns are reported: reference sample-identifier ("sample_id"); amount of reads in fastq files before pre-processing analysis ("raw_fastq"); amount of reads in fastq files after adapter trimming and reads quality filtering ("trimmed_fastq"); amount of reads after contaminants remotion ("decotaminated_fastq"). In the panel "Alignment_Post-processing", the following columns are reported: reference sample identifier ("Sample_id"); amount of paired-reads used as input for genome (GRCh38) alignment procedure ("Reads used as input for alignment"); total amount of fragments mapped to genome ("Total_mapped_reads"); amount of fragments with un-ambiguous mapping position in genome ("Uniquely mapped reads"); amount of properly-paired reads after deduplication, and mulit-mapping remotion procedures ("Dedupuplicated_properly-paired_no-multimapped_reads"). In the panel "Selected_m6A_Peaks" columns are reported as in standard bed12 files.

File Name: Supplementary_Data _3_FUSP525L_HITS_CLIP.xls
Description: Summary of HITS-CLIP analysis. In the panel "Description" all the information about the other panels is reported. In the panel "Pre-processing", the following columns are reported: reference sample-identifier ("sample_id"); amount of reads in fastq files before pre-processing analysis ("raw_fastq"); amount of reads in fastq files after adapter trimming and reads quality filtering ("trimmed_fastq"); amount of reads after contaminants remotion ("decotaminated_fastq").
In the panel "Alignment_Post-processing", the following columns are reported: reference sample identifier ("Sample_id"); amount of paired-reads used as input for genome (GRCh38) alignment procedure ("Reads used as input for alignment"); total amount of fragments mapped to genome ("Total_mapped_reads"); amount of fragments with un-ambiguous mapping position in genome

(“Uniquely mapped reads”); amount of properly-paired reads after deduplication, and mulit-mapping remotion procedures (“Dedupuplicated_properly-paired_no-multimapped_reads”). In the panel “Selected_HITSCLIP_Peaks” columns are reported as in Standard bed6 format with genomic coordinates of significat peaks.

File Name: Supplementary_Data 4_oligonucleotides.xlsx
Description: List of oligonucleotides used in this study. In the panel “PCR primers” the sequences of the oligonucleotides used for PCR in this study are reported. The sequences of the smFISH probes used for HUWE1, CLSTN1 and CALM1 are reported in the panel named as “HUWE1 mFISH probes”; “CLSTN1 mFISH probes”, “CALM1 mFISH probes”, respectively.

File Name: figures_supplementary.pdf
Description: Supplementary Figures 1-4.

File Name: Source data.xlsx
Description: Raw data for any graphs, plots, and uncropped versions of any gels or blots presented both in the main and supplementary figures. Each panel is named as the related figure.
