# Supplementary material for: M6A reduction relieves FUS-associated ALS granules
Source: Nat Commun. 2024 Jun 12;15:5033. doi: 10.1038/s41467-024-49416-5 (PMC11169559; doi:10.1038/s41467-024-49416-5)
Supplement: Supplementary file 8 — Reporting Summary [file 41467_2024_49416_MOESM8_ESM.pdf]

nature portfolio

Corresponding author(s): Irene Bozzoni

Last updated by author(s): 2024/05/02

Reporting Summary

Nature Portfolio wishes to improve the reproducibility of the work that we publish. This form provides structure for consistency and transparency in reporting. For further information on Nature Portfolio policies, see our Editorial Policies and the Editorial Policy Checklist.

Statistics

For all statistical analyses, confirm that the following items are present in the figure legend, table legend, main text, or Methods section.

n/a	Confirmed	
☐	☒	The exact sample size (*n*) for each experimental group/condition, given as a discrete number and unit of measurement
☐	☒	A statement on whether measurements were taken from distinct samples or whether the same sample was measured repeatedly
☐	☒	The statistical test(s) used AND whether they are one- or two-sided<br>*Only common tests should be described solely by name; describe more complex techniques in the Methods section.*
☒	☐	A description of all covariates tested
☐	☒	A description of any assumptions or corrections, such as tests of normality and adjustment for multiple comparisons
☐	☒	A full description of the statistical parameters including central tendency (e.g. means) or other basic estimates (e.g. regression coefficient) AND variation (e.g. standard deviation) or associated estimates of uncertainty (e.g. confidence intervals)
☐	☒	For null hypothesis testing, the test statistic (e.g. *F*, *t*, *r*) with confidence intervals, effect sizes, degrees of freedom and *P* value noted<br>*Give P values as exact values whenever suitable.*
☒	☐	For Bayesian analysis, information on the choice of priors and Markov chain Monte Carlo settings
☒	☐	For hierarchical and complex designs, identification of the appropriate level for tests and full reporting of outcomes
☐	☒	Estimates of effect sizes (e.g. Cohen's *d*, Pearson's *r*), indicating how they were calculated

Our web collection on statistics for biologists contains articles on many of the points above.

Software and code

Policy information about availability of computer code

Data collection	Agarose gels and western blots were acquired thanks to a ChemiDoc™ MP Imager (Bio-Rad). DNA amplification in RT-qPCR was monitored with an Applied Biosystems™ 7500 Fast or StepOnePlus System qPCR instrument. Images for transwell-migration assay were acquired using inverted confocal Olympus IX73 microscope equipped with a Crestoptics X-LIGHT V3 spinning disk system and a Prime BSI Express Scientific CMOS camera and with an Olympus iX83 FluoView1200 laser scanning confocal microscope. For spot-variation FCS measurements a custom laser-scanning microscope equipped with a 5x5 single-photon avalanche diode (SPAD) array detector was used. All RNA-seq samples were sequenced on an Illumina Novaseq 6000 Sequencing system.
Data analysis	Image Lab™ 5.2.1 Software (BioRad) was used for the acquisition and analysis of agarose gels and western blots. DNA amplification in RT-qPCR was analyzed with the Applied Biosystems™ 7500 Fast or the StepOnePlus System qPCR instrument related software. Images were acquired using the MetaMorph software (Molecular Devices) and analyzed with FiJi software . For bioinformatic analyses several tools were used including Trimmomatic, Cutadapt, BWA-MEM, CIRI2, edgeR, HTSeq, STAR, SAMtools 1.10, Bioconductor, MACS version 2.2.7.1, Picard suite (v2.24.1) , OmniCLIP software (v0.2.0), WebGestalt R tool (v0.4.4), deepTools suite, bigwigCompare function (v3.5.1) . When needed data were further processed with Microsoft Excel.

For manuscripts utilizing custom algorithms or software that are central to the research but not yet described in published literature, software must be made available to editors and reviewers. We strongly encourage code deposition in a community repository (e.g. GitHub). See the Nature Portfolio guidelines for submitting code & software for further information.

Data

Policy information about availability of data

All manuscripts must include a data availability statement. This statement should provide the following information, where applicable:

- Accession codes, unique identifiers, or web links for publicly available datasets
- A description of any restrictions on data availability
- For clinical datasets or third party data, please ensure that the statement adheres to our policy

All software, links to websites or tools used for this work are referred to in the methods section or in the figure legends. Additional dedicated scripts developed for this work are available upon request. High throughput sequencing data reported in this paper are deposited at GEO (GSE242771, Token: gvidaeyyttidvoj). Source data are provided with this paper.

Research involving human participants, their data, or biological material

Policy information about studies with human participants or human data. See also policy information about sex, gender (identity/presentation), and sexual orientation and race, ethnicity and racism.

Reporting on sex and gender	not applicable
Reporting on race, ethnicity, or other socially relevant groupings	not applicable
Population characteristics	not applicable
Recruitment	Human primary fibroblasts were obtained from a patient carrying the p.R518I in FUS gene.
Ethics oversight	Human primary fibroblasts were obtained from a patient carrying the p.R518I in FUS gene, after written informed consent and approval of the Ethic Committee (Protocol nr. A.133/CE/2013).

Note that full information on the approval of the study protocol must also be provided in the manuscript.

Field-specific reporting

Please select the one below that is the best fit for your research. If you are not sure, read the appropriate sections before making your selection.

☒ Life sciences ☐ Behavioural & social sciences ☐ Ecological, evolutionary & environmental sciences

For a reference copy of the document with all sections, see nature.com/documents/nr-reporting-summary-flat.pdf

Life sciences study design

All studies must disclose on these points even when the disclosure is negative.

Sample size	Sample size was chosen based upon previous studies in the field, the technical difficulty and throughput of the assay. The sample size (n) of each experiment is indicated in the figure legends in the main manuscript and supplementary information files. At least 3 biological replicates was used in this study.
Data exclusions	No data points were excluded fron analysis in any experiment depicted in this manuscript.
Replication	Every experiment was performed multiple times to ensure that the findings in this paper were remarkably reproducible.
Randomization	For cell culture based experiments cells were split from the same batch of cells and randomly divided for each treatment in each biological replicate.
Blinding	The investigators were not blinded to allocation during experiments and outcome assessment.

Reporting for specific materials, systems and methods

We require information from authors about some types of materials, experimental systems and methods used in many studies. Here, indicate whether each material, system or method listed is relevant to your study. If you are not sure if a list item applies to your research, read the appropriate section before selecting a response.

Materials & experimental systems

n/a	Involved in the study
☐	☒ Antibodies
☐	☒ Eukaryotic cell lines
☒	☐ Palaeontology and archaeology
☒	☐ Animals and other organisms
☒	☐ Clinical data
☒	☐ Dual use research of concern
☒	☐ Plants

Methods

n/a	Involved in the study
☒	☐ ChIP-seq
☒	☐ Flow cytometry
☒	☐ MRI-based neuroimaging

Antibodies

Antibodies used

Anti-FUS mouse antibody (Santa Cruz Biotechnology, #sc-47711, 1:300
Anti-METTL3 rabbit monoclonal antibody (Abcam, #ab195352, 1:1000)
Anti-METTL14 polyclonal antibody (Atlas, #HPA038002, 1:1000)
Anti-Flag M2-Peroxidase (HRP) (Sigma-Aldrich, #A8592, 1:2500)
Anti-ACTB-Peroxidase (AC-15) monoclonal antibody (Sigma-Aldrich, #A3854, 1:2500)
Anti-GAPDH (6C5) monoclonal antibody (Santa Cruz Biotechnology, #sc-32233, 1:1000)
Anti-Rabbit IgG (H+L) Secondary Antibody, HRP (Thermo Fisher Scientific, #31460, 1:10000)
Anti-Mouse IgG (H+L) Secondary Antibody, HRP (Thermo Fisher Scientific, #32430, 1:10000)
Anti-Actinin monoclonal antibody (Santa Cruz Biotechnology, #sc-390205, 1:1000)
Anti-ALKBH5 monoclonal antibody (Proteintech, #67811-1-Ig, 1:1000)
Anti-HPRT (HRP) antibody (Santa Cruz Biotechnology, #sc-20975, 1:1000)
anti-G3BP1 rabbit antibody (1:300, ab181150 Abcam)
anti-FLAG mouse antibody (1:400, F1804 Sigma-Aldrich)
anti-Beta III Tubulin (TUJ1) chicken Antibody (1:400, AB9354 Sigma-Aldrich)
goat anti-rabbit Alexa Fluor 488 (1:300, A11008 Thermo Fisher Scientific),
goat anti-mouse Alexa Fluor 488 (1:300, A11001 Thermo Fisher Scientific)
goat anti-rabbit Alexa Fluor 555 (1:300, 111-165-003 Jackson Immunoresearch)
goat anti-mouse Alexa Fluor 555 (1:300, 115-165-003 Jackson Immunoresearch)
goat anti-chicken Alexa Fluor 594 (1:300, ab150176 Abcam)
donkey anti-mouse Alexa Fluor Plus 647 (1:300, A32787 Thermo Fisher Scientific)
donkey anti-rabbit Alexa Fluor Plus 647 (1:300, A32795 Thermo Fisher Scientific)

Validation

All of the antibodies used in this study were commercial and suitable for specific purposes. The antibodies were validated based on the information from the manufacturer's instructions and was supported by multiple publications.

Eukaryotic cell lines

Policy information about cell lines and Sex and Gender in Research

Cell line source(s)

SK-N-BE cells were obtained from the ATCC bank (CRL-2271).
Human NIL iPSCs were kindly provided by Prof. Alessandro Rosa (Garone et al. 2018).
Human primary fibroblasts were obtained from a patient carrying the p.R518I in FUS gene, after written informed consent and approval of the Ethic Committee (Protocol nr. A.133/CE/2013).

Authentication

SK-N-BE cells were authenticated by ATCC. No information is available about the authentication of human fibroblasts. Human NIL iPSCs were previously authenticated as described in Garone M. et al., 2018.

Mycoplasma contamination

Cells were tested and resulted negative for Mycoplasma contamination.

Commonly misidentified lines
(See ICLAC register)

No commonly misidentified cell lines were used in this study.

Plants

Seed stocks

Report on the source of all seed stocks or other plant material used. If applicable, state the seed stock centre and catalogue number. If plant specimens were collected from the field, describe the collection location, date and sampling procedures.

Novel plant genotypes

Describe the methods by which all novel plant genotypes were produced. This includes those generated by transgenic approaches, gene editing, chemical/radiation-based mutagenesis and hybridization. For transgenic lines, describe the transformation method, the number of independent lines analyzed and the generation upon which experiments were performed. For gene-edited lines, describe the editor used, the endogenous sequence targeted for editing, the targeting guide RNA sequence (if applicable) and how the editor was applied.

Authentication

Describe any authentication procedures for each seed stock used or novel genotype generated. Describe any experiments used to assess the effect of a mutation and, where applicable, how potential secondary effects (e.g. second site T-DNA insertions, mosiacism, off-target gene editing) were examined.
